# Mycobacterium abscessus Complex Identification with Matrix-Assisted Laser Desorption Ionization–Time of Flight Mass Spectrometry

**DOI:** 10.1128/JCM.00494-15

**Published:** 2015-06-18

**Authors:** Theofano Panagea, David H. Pincus, Dorothy Grogono, Melissa Jones, Josephine Bryant, Julian Parkhill, R. Andres Floto, Peter Gilligan

**Affiliations:** a Sismanogleio-A.Flemig General Hospital, Athens, Greece; b University of North Carolina at Chapel Hill, Chapel Hill, North Carolina, USA; c bioMérieux, Inc., Hazelwood, Missouri, USA; d Cambridge Centre for Lung Infection, Papworth Hospital, Cambridge, United Kingdom; e University of Cambridge, Cambridge, United Kingdom; f UNC Health Care, Chapel Hill, North Carolina, USA; g Wellcome Trust Sanger Institute, Hinxton, United Kingdom; h Cambridge Institute for Medical Research, University of Cambridge, Cambridge, United Kingdom

## Abstract

We determined that the Vitek MS Plus matrix-assisted laser desorption ionization–time of flight mass spectrometry using research-use-only (RUO) v.4.12 and *in vitro*-diagnostic (IVD) v.3.0 databases accurately identified 41 Mycobacterium abscessus subsp. abscessus and 13 M. abscessus subsp. massiliense isolates identified by whole-genome sequencing to the species but not the subspecies level, from Middlebrook 7H11 and Burkholderia cepacia selective agars. Peak analysis revealed three peaks potentially able to differentiate between subspecies.

## TEXT

Nontuberculous mycobacteria (NTM) are emerging pathogens with increasing prevalence in clinical settings in the industrialized world (1). Mycobacterium abscessus is emerging as a significant respiratory pathogen accounting for a large proportion of NTM disease in cystic fibrosis (CF) patients (2–6). They also cause a variety of infections in non-CF patients, such as chronic pulmonary, skin and soft tissue, and postsurgical infections (7).

Recent evidence (8, 9) supported by whole-genome sequencing (WGS) data (2) suggest that the M. abscessus complex comprises three subspecies M. abscessus subsp. abscessus (sensu stricto), M. abscessus subsp. massiliense, and M. abscessus subsp. bolletii although the taxonomy of this complex of organisms is debated. Discrimination between them is clinically important due to the differences in resistance and treatment response rates (1, 7, 10–14) and relies on the sequencing of multiple genes such as *erm*(41), 23S rRNA, *hsp65*, and *rpoB* (11, 12, 15), which delays the result and is expensive and time-consuming to perform in a routine clinical microbiology laboratory.

Matrix-assisted laser desorption ionization–time of flight mass spectrometry (MALDI-TOF MS) has been evaluated in several studies, using different systems and different extraction protocols, as an inexpensive, rapid, and efficient method in the identification of mycobacteria (16–19).

In the present study, using a previously evaluated extraction protocol (20), we determined the performance of two MALDI-TOF MS databases for the identification of M. abscessus isolates using a culture collection of M. abscessus isolates uniquely characterized by WGS (2). Two solid media used in our laboratory to isolate M. abscessus from clinical specimens, Middlebrook 7H11 and Burkholderia cepacia selective agar (BCSA), were evaluated. We have previously shown that M. abscessus can be recovered on BCSA from routine bacterial respiratory cultures of CF patients (21). We also assessed the influence of culture age on the MALDI-TOF identifications and the ability of the system to identify isolates at the subspecies level and to detect genetic relatedness among them.

Fifty-four mycobacterial clinical strains, 41 M. abscessus subsp. abscessus and 13 M. abscessus subsp. massiliense isolated between May 2005 and March 2013 in the Clinical Microbiology/Immunology Laboratories at University of North Carolina Health Care were initially identified as M. abscessus by sequential 16S rRNA and *hsp65* gene sequencing (22, 23). Isolates were identified to the subspecies level by WGS, which was followed by phylogenetic analysis (data not shown), each performed at the Wellcome Trust Sanger Institute (Hinxton, United Kingdom) as described elsewhere (2). Mycobacterial isolates were recovered from respiratory samples of CF patients (*n* = 43) and a variety of samples were from non-CF patients, sputum (*n* = 4), bronchial aspirate (*n* = 1), wound (*n* = 3), abscess (*n* = 1), biopsy specimen (*n* = 1), and blood (*n* = 1).

Isolates were subcultured on Middlebrook 7H11 (BD Diagnostic Systems, Sparks, MD) and BCSA plates (Remel Microbiology Products; Thermo Scientific, Lenexa, KS) and incubated for an equal amount of time, 3 to 4 days depending on growth rate, at 35°C with 5% CO_2_. The bioMérieux extraction protocol reported on by Mather and colleagues was followed (20). The extracted isolates were tested using the Vitek MS Plus system (bioMérieux SA, Marcy l'Etoile, France) according to manufacturer's recommendations (20). Each extracted isolate was tested twice to avoid acquisition errors. Escherichia coli ATCC 8739 was included in each run as a positive control. Generated spectra were automatically compared to the research-use-only (RUO) Saramis Knowledge Base (bioMérieux SA) database v.4.12 with the use of Saramis Premium software as well as the draft (development) *in vitro*-diagnostic (IVD) v.3.0 database with the use of an R&D software (SpectraIdentifier R2.1.0), and the identification confidence scores (IDCSs) were recorded. IDCS is the identification confidence level for the best match to the database. Unidentified isolates were considered strains on double testing and individual spectra that gave an IDCS of <75% against the RUO Saramis Knowledge Base and more than four matches against the IVD v.3.0 database. For those isolates where no matches were found, extraction and testing were repeated.

All 54 isolates were accurately identified as M. abscessus by the Vitek MS Plus system with each database and each medium, except one isolate on Middlebrook 7H11 and one on BCSA against RUO Saramis Knowledge Base, for which M. abscessus was nevertheless the closest match. Extraction was repeated for one isolate that yielded no match with either database. Average IDCSs were 91.4% and 93.7% (*P* = 0.062) for Middlebrook 7H11 and BCSA, respectively, against the RUO v.4.12 database and 99.9% against the IVD v.3.0 database for each medium. Out of 23 individual spectra that yielded no identification with the RUO v.4.12 database, only two remained unidentified when the IVD v.3.0 database was used for comparison.

Databases available at the time of this study did not discriminate at the subspecies level. Our results are consistent with previous reports using either the Biotyper (Bruker Daltonics) (17–20) or Vitek MS system (bioMérieux SA) (20, 24) despite the differences in the isolation media, extraction protocol, and databases used.

In order to assess the effect of culture age on the system's performance, 10 phylogenetically distinct isolates, 7 M. abscessus subsp. abscessus and 3 M. abscessus subsp. massiliense, were tested from Middlebrook 7H11 and BCSA after 72 h, 96 h, and 120 h of incubation. Each strain was subcultured on the above media on three consecutive days to simultaneously yield cultures at all three growth stages and include them in the same run in order to avoid inter-run variation.

**FIG 1:**
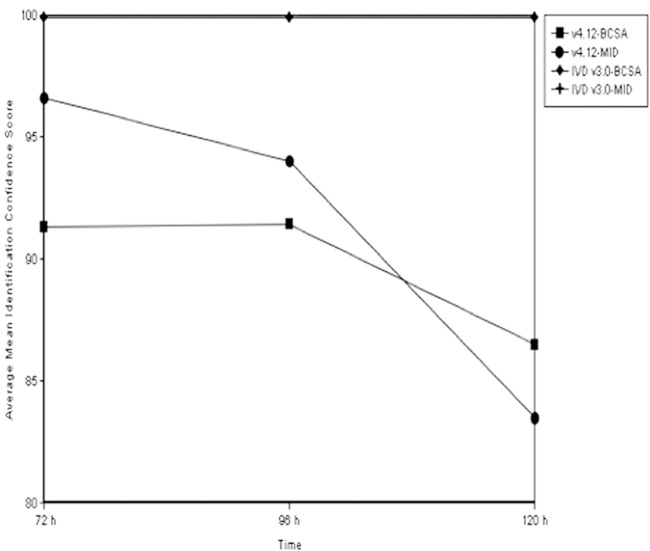
Effect of culture age on MALDI-TOF MS performance using the RUO v.4.12 and IVD v.3.0 databases. The average mean IDCS decreased significantly at 120 h compared to 72 h and 96 h for each medium. When the IVD v.3.0 database was used, no decrease was observed at all time points or in either medium.

Although strains were successfully identified as M. abscessus at all time points, the average mean IDCS with the RUO v.4.12 database decreased significantly at 120 h compared to 72 h and 96 h for each medium (repeated measure analysis, *P* < 0.05). In detail, the score decreased from 96.6% and 91.3% at 72 h and 94% and 91.4% at 96 h to 83.5% and 86.5% at 120 h for 7H11 and BCSA, respectively (Fig. 1). Our data are in accordance to the results of Mather et al. (20) who also observed a decline on identification confidence scores with the progression of culture age, possibly attributed to differences in the colony age used for database creation, which one can overcome by lowering the threshold used for identification. No decline in IDCS was observed for either medium when the IVD v.3.0 database was used; all IDCS were 99.9% at all time points due to the different algorithm used for the construction of the IVD v.3.0 database. Nevertheless, it is preferable to test colonies at a younger stage of growth, as soon as one is able to distinguish between different morphotypes, in order to achieve the closest match to the existing databases.

Finally, a dendrogram was created with the isolates' spectra generated from Middlebrook 7H11 using Saramis Premium software. Neither strain belonging to the same subspecies nor genetically related ones clustered separately (data not shown), suggesting an inability to use the Saramis algorithm for automated identification of these two subspecies or for detection of their clonality. Nevertheless, subsequent peak analysis (performed manually using ±0.08% tolerance to define peak equivalence, also used by the Saramis software) revealed three peaks differentially present among the two subspecies, averaged at 8,508.66 *m/z*, 9,473.31 *m/z*, and 8,769.01 *m/z*. In detail, peak 8,508.66 *m/z* was present in 11/13 (84.6%) M. abscessus subsp. massiliense and absent from 39/41 (95.1%) M. abscessus subsp. abscessus isolates, peak 9,473.31 *m/z* was absent from all 13 M. abscessus subsp. massiliense and present in only 7/41 (17%) M. abscessus subsp. abscessus isolates, and peak 8,769.01 *m/z* was absent from all 41 M. abscessus subsp. abscessus and present only in 5/13 (38.4%) M. abscessus subsp. massiliense isolates (Table 1). We speculate that the last two peaks are identical with the ones generated by the Bruker Biotyper at 9,477.48 *m/z* and 8,771.73 *m/z* published by Teng et al. (25) and reported to be 100% specific for M. abscessus subsp. abscessus and M. abscessus subsp. massiliense, respectively. Additionally, peaks published by Fangous et al. (26) were also evaluated, and the only peaks observed were the ones at 2,081 *m/z* and 3,378 *m/z*. The 2,081 *m/z* peak was found to be less sensitive and specific than reported, as it was not uniformly present in M. abscessus subsp. abscessus (13/41) while also present in 1/13 M. abscessus subsp. massiliense, whereas the 3,378 *m/z* peak was—similarly to the Fangous et al. (26) publication—present in all isolates of the two subspecies. Unfortunately, we are unable to evaluate its use on M. abscessus subsp. bolletii identification since such isolates were not found in our patient population and analyzed by WGS.

**TABLE 1:** Differential presence of three peaks in spectra generated by MALDI-TOF MS among subspecies of the M. abscessus complex

Peak avg mean (*m/z*)	Peak range (*m/z*)	No. (%) present in M. abscessus subsp. abscessus	No. (%) present in M. abscessus subsp. massiliense	Subspecies
8,508.66	8,506.29–8,511.80	2 (4.9)	11 (84.6)	massiliense
9,473.31	9,471.37–9,477.34	7 (17)	0 (0)	abscessus
8,769.01	8,767.94–8,771.16	0 (0)	5 (38.4)	massiliense

Identification of the different subspecies is important in the clinical environment. Clarithromycin, used for the treatment of these infections (6, 10), is more effective against M. abscessus subsp. massiliense lung infections, while resistance is common in M. abscessus subsp. abscessus and M. abscessus subsp. bolletii isolates (11–13, 27). Additionally, there is differential susceptibility to various antimycobacterial drugs (1, 14). Our data, although insufficient due to the lack of M. abscessus subsp. bolletii isolates, enhance the already existing literature, which suggests the potential of MALDI-TOF MS to identify subspecies within the M. abscessus complex.

In conclusion, MALDI-TOF MS is a powerful technique capable of accurate and rapid identification of M. abscessus isolates not only from specific mycobacterial culture media but also from selective media used to detect B. cepacia complex isolates in routine bacterial cultures of CF patients. The IVD v.3.0 database provides excellent results on M. abscessus identification, minimizing unidentified results compared to RUO Saramis Knowledge Base v.4.12. A current potential shortcoming of each bioMérieux database for M. abscessus evaluated in this study is that they were generated using isolates (18 for RUO Saramis Knowledge Base v4.12 and 20 for IVD v3.0) of M. abscessus identified only to the species level. The inability to automate identification to the subspecies level then was expected. However, MALDI-TOF technology is sometimes inadequate to differentiate at the subspecies level even when reference identification to subspecies is included in the database. As molecular tools are applied to further unravel the taxonomy of a variety of emerging human bacterial and fungal pathogens, an evolution of MALDI-TOF databases will be required. Studies such as this one using WGS as the reference method demonstrate the current shortcomings but also the promise of this technology.
